# The punctuated evolution of the Venusian atmosphere from a transition in mantle convective style and volcanic outgassing

**DOI:** 10.1126/sciadv.adn9861

**Published:** 2025-01-10

**Authors:** Matthew B. Weller, Walter S. Kiefer

**Affiliations:** ^1^ Department of Earth and Environmental Sciences, Jonsson-Rowland Science Center, Rensselaer Polytechnic Institute, Troy, NY, USA.; ^2^ Lunar and Planetary Institute/USRA, Houston, TX, USA.

## Abstract

A key question in the planetary sciences centers on the divergence between the sibling planets, Venus and Earth. Venus currently does not operate with plate tectonics, and its thick atmosphere has led to extreme greenhouse conditions. It is unknown if this state was set primordially or if Venus was once more Earth-like. Here, we explore Venus as an example of a planet that recently transitioned between tectonic regimes. Our results show that transitions naturally lead to substantial resurfacing and melt-generated outgassing from lithosphere-breaking events and overturns, with 3 to 10 bars of atmosphere generated per overturn over ~60–million year timescales and ~10 to 100 bars outgassed over billion-year time frames. We find that the observation of Venus with a thick greenhouse atmosphere and the inferences of currently low volcanic rates and previous prodigious volcanic rates are consistent with a planet that has undergone a transition in tectonics, suggesting that Venus once hosted clement surface conditions and was more Earth-like.

## Teaser

Venus' current surface and atmosphere were caused by a transition in tectonics states, suggesting a once more Earth-like Venus..

## INTRODUCTION

Venus is the least understood of the terrestrial planets. Despite broad similarities in mass, size, and chemical composition to the Earth [e.g., (*1*)], Venus’ atmosphere and surface are notably different. Venus is blanketed by a thick ~92-bar atmosphere that is dominated by ~89 bars of CO_2_, with the remainder made up of N_2_ and trace amounts of other gases (*2*, *3*). The atmospheric reservoir of carbon for Venus accounts for ~2.2× the total surficial carbon inventory for the Earth (*4*), which has resulted in an extreme greenhouse state with surface temperatures of ~740 K (*5*). Vast volcanic plains emplaced within the past 300 million years (Myr) to 1 billion years (Gyr) (*6*–*8*), encompassing ~80% of Venus’ surface, are suggestive of relatively recent and prodigious melting events. Much of Venus’ surface record is obscured by this relatively recent volcanism, leading to uncertainty in Venus’ climatic, atmospheric, and tectonic past (*9*–*16*). Volcanism, however, is intimately linked to atmosphere generation and tectonic states [e.g., (*9*, *12*)], and the atmosphere can be viewed as long-lived record of the planet’s surface and interior evolution [e.g., (*12*)]. Therefore, exploring the linked evolution of the atmosphere, surface, tectonics, and interior is critical for understanding observations of Venus today, as well as constraining the distant Venusian past.

Currently, Venus shows no clear evidence of Earth-like plate tectonic activity (*17*, *18*). However, there are several lines of evidence that suggest that Venus once did have a mobile lithosphere perhaps not dissimilar to Earth. The region of Western Ishtar Terra likely formed through convergence and is often considered an analog to the Himalayas and Tibetan Plateau on Earth (*19*–*21*). Furthermore, active subduction beneath the margins of Artemis and Quetzalpetlatl coronae has been proposed based on gravity and morphological observations (*22*). These observations, along with general inferences of current limited large-scale extension (*17*, *23*–*25*), are consistent with suggestions of some form of episodic lid or transitional tectonic regime for Venus (*10*, *11*, *18*, *26*–*29*), indicating that Venus may be an example of a transition in tectonic regimes [e.g., (*10*–*12*)].

Although we have limited measurements of the current atmosphere and climate of Venus [e.g. (*5*)], the planet’s atmospheric and climatic evolution is largely unconstrained [e.g., (*9*)]. Possible scenarios range from an effectively static view of the atmosphere that suggests that Venus’ current greenhouse state was set primordially [e.g., (*15*, *16*)] to the idea that Venus was once much wetter and more Earth-like and thus potentially habitable, with the current atmosphere the result of a great climate transition [e.g., (*14*, *30*, *31*)]. Despite observations of a relatively quiescent tectonic Venus, there is indirect and sometimes speculative evidence of volcanic activity at differing temporal scales at the surface [e.g., (*32*–*39*)], suggesting outgassing to the atmosphere within the recent geologic past. Although the specific timing of the emplacement of the Venusian atmosphere is unclear, it is increasingly apparent that the evolution of the atmosphere can influence the style of mantle convection of the planet [e.g., (*10*, *11*)], suggesting that the evolution of the atmosphere, surface, tectonics, and interior is linked and is critical for understanding current observations of Venus.

The goal of this work is to determine the effects of a climate-driven transition in tectonic regimes on the coupled tectonic, mantle, and atmospheric systems of Venus and evaluate whether observations of Venus today are consistent with a more Earth-like and potentially habitable Venus in the past. To explore this potential evolutionary pathway for Venus, our approach is to leverage recent advances in our understanding of convective physics using coupled thermal-tectonic three-dimensional (3D) numerical experiments [e.g., (*11*)], which allow for self-consistent changes in tectonic states that cannot be explored with simpler models. Consistent with the potential of a more mobile and perhaps clement past [e.g., (*14*, *30*, *31*)], the starting condition of all of our numerical experiments are assumed to be Earth-like in surface temperatures, internal structure, lithospheric yield strengths, and tectonic state. We allow for changes in governing parameters such as surface temperatures and lithospheric yield strengths that serve to usher in a change in global tectonics (details in the next section and in Methods), couple these results to gas speciation models (*40*), and explore the outgassing evolution of Venus’ atmosphere. We find that Venus today may be consistent with a transition from an early plate tectonic–like mobile lid into a stagnant lid state and that Venus’ atmosphere may have evolved through punctuated episodic (multibar atmosphere generation events) over the past couple gigayears.

## RESULTS

### Tectonic evolution and oscillations

**Fig. 1.**
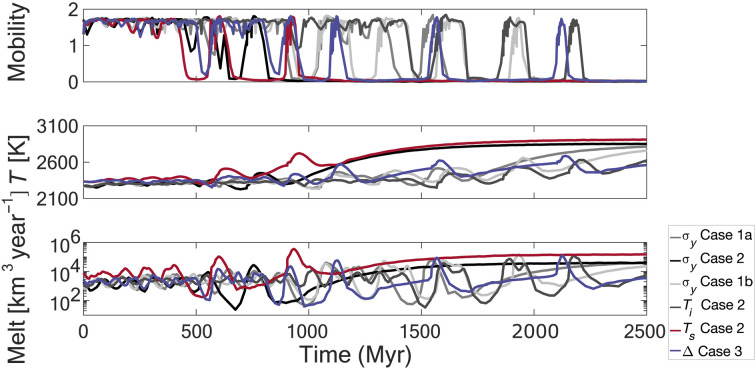
Oscillatory behavior of transitions in tectonic regimes from a subset of coupled thermal-tectonic numerical experiments. Each experiment initiates in a mobile (plate tectonic–like) regime at time 0 and transitions to a stagnant (single-plate) regime over time. Quantities with [ ] denote dimensional values. The top panel indicates the ratio of surface to internal velocities or mobility. Mobility ≥ ~1 indicates horizontal motions and a mobile surface (mobile lid; or plate tectonic–like). Mobility ≤ 0.1 indicates surface immobility and quiescence (stagnant lid; or single plate). The middle panel shows internal mantle temperatures. The bottom panel is the melt production rate, which records melt generated within the uppermost mantle. Cases are outlined in Table 1. The nonadiabatic temperature contrast is 3000 K for all cases.

If Venus once supported more clement conditions and some form of surface mobility, then a transition in the global style of tectonics would be expected from physical arguments and observations. One mechanism to affect a transition is through the loss of liquid water from the surface [e.g., (*41*)] and, consequently, pore fluids, which would strengthen faults (*42*) and increase the effective global yield strength, resisting surface motions associated with mantle convection. Another mechanism to affect a tectonic transition occurs through an increase in surface temperatures, which serve to reduce the buoyancy forces that drive motion of the lithosphere (*10*, *11*, *43*–*46*). Recent advances in the understanding of the convective physics of regime transitions have shown that 3D simulations of planetary mantle evolution are uniquely able to capture the complex and 3D nature of a regime transition [e.g., (*10*, *44*, *47*, *48*)]. 1D parameterized models are incapable of exploring this because a transition in tectonic states violates fundamental model assumptions and because any such transition is, by definition, imposed rather than determined by model physics [e.g., (*12*, *45*)]. A 2D model cannot capture the spatially complex dynamics exhibited by the 3D system (*11*, *44*, *47*). In this work, we examine 13 fully 3D spherical numerical experiments to explore the effects of both changing global yield strengths and surface temperatures, which are divided into four distinct categories to account for uncertainty in Venus’ surface and interior evolution (summarized in Table 1): (i) yield strength variations potentially brought on by an early phase of water loss and dehydration of the surface (σ*_y_* cases; 135 to 162 MPa); (ii) initial temperature variations from higher internal heating rates [*T_i_* Cases; 3 to 6% enrichment in radiogenic heating rate (*Q*; where *Q* is defined as the nondimensional heating rate, see Eq. 5 in Methods)]; (iii) surface temperature variations from early hothouse or greenhouse conditions (*T_s_* Cases; 273 to 723 K); and (iv) depleting levels of radiogenic elements with an increase in surface temperatures and yield strengths (∆ cases; up to a 30% depletion in radiogenic heating rate). A subset of cases was also considered with a different starting condition to emulate the effects of unconsidered perturbations (“noise”) in the system on the dynamics of transition [see (*45*) for details]. Our numerical results indicate that, from a conditionally stable mobile lid (here identified at *Q* = 60, *T_s_* = 273 K, and σ*_y_* = 135 MPa), a transition in tectonic regimes is ushered in by as little as an 8% increase in fault strength or a 10% increase in the surface temperature from this condition. A representative subset of transitioning systems, from plate tectonic–like mobile lids to single-plate stagnant lids, is shown in Fig. 1.

**Table 1.** Numerical cases explored and atmospheric pressures generated. The yield strength is dimensionalized using the same approach and parameters as (*11*). Surface temperatures are dimensionalized using the total temperature contrast of 3000 K. The internal heat production (*Q*) is chosen to emulate a planetary system with high internal temperatures (here *Q* = 60). The initial state yield strength and surface temperatures are set a: σ*_y_* = 1.00 × 10^5^; 135 MPa and *T_s_* = 0; 273 K, nondimensional and dimensional, respectively. Values marked with Δ indicate a change in the associated parameter.

Name	∆σ_*y*_ (nondim)	∆σ_*y*_ (MPa)	∆*T*_*s*_ (nondim)	∆*T*_*s*_ (K)	∆*Q* (nondim)	Venus *P*_atmo_ (%)	# overturns
σ*_y_* Case 1a	0.08 × 10^5^	11	0	0	0	22	3
σ*_y_* Case 1b	0.08 × 10^5^	11	0	0	0	43	5
σ*_y_* Case 2	0.10 × 10^5^	14	0	0	0	19	3
*T_i_* Case 1	0	0	0	0	1.5	37	3
*T_i_* Case 2	0	0	0	0	3	56	4
*T_s_* Case 1a	0	0	0.01	30	0	71	5
*T_s_* Case 1b	0	0	0.01	30	0	52	4
*T_s_* Case 2	0	0	0.03	90	0	32	2
*T_s_* Case 3	0	0	0.05	150	0	306	2
∆ Case 1	0	0	0.15	450	−7	35	3
∆ Case 2	0	0	0.15	450	−15	23	3
∆ Case 3	0	0	0.15	450	−23	33	5
∆ Case 4	0.20 × 10^5^	27	0.15	450	−30	20	3

We previously showed that transitions in regimes are governed by both regional- and global-scale instabilities (*11*). This results in punctuated and extreme oscillations in key system observations, e.g., internal temperatures and magma production rates (see Fig. 1 caption for descriptions). In the initial plate tectonic–like mobile regime (<400 Myr for all models), melt is generated by passive upwelling along relatively stable yielding zones, which are analogous to plate boundaries. As the system destabilizes, it enters a transitional (or episodic) state (spikes and plateaus of activity after 400 Myr; Fig. 1). The transitional regime is characterized by extreme oscillations in surface mobility, interior temperature, and melt production. The associated changes in convective circulation create hemispheric- to subhemispheric-scale upwelling structures or plumes, within which large-scale decompression melting causes most of the melting for the system (*11*). For each of the cases considered, overturns remain restricted to this scale and do not destabilize the entire lithosphere. This is inferred to be the result of hemispheric-scale upwellings (plumes) destabilizing the lithosphere. Plumes are transient and stochastic features, and over time, they weaken and shut off, only for plumes to initiate in differing regions. Consequently, and following from the results in (*11*), different overturn events and induced melting are localized to differing portions of the planet. With sufficient overturn events, areas that originally experienced extensive melting may again be affected stochastically. During individual overturn events, melt production increases by a factor of O (15) at peak activity [where O () indicates order of magnitude] and then decreases by a factor of O (100) during inactivity relative to the baseline plate tectonic–like mobile state. Collectively, the interval from a period of extreme yielding and melting to the cessation of both yielding and melting is termed an overturn phase. As the system further evolves, punctuated increases in mobilities (overturn phases) cease as yielding ceases, and the system enters its final quiescent stagnant state. Initially, melt production remains low, comparable to the inactive periods of the transitional regime. At ~100 Myr after yielding cessation, reduction in the heat flow through the thickened lithosphere causes both internal temperatures and melt generation to begin to increase. After ~600 Myr, both internal temperatures and melting plateau and reach steady-state values. During this phase, melt becomes spatially diffuse and increases by a factor of O (7) relative to the baseline plate tectonic–like mobile state.

The timing of the onset of oscillations and subsequent surface immobility in large part depends on the strength of the initial perturbation (Fig. 1). The transition itself is highly dynamic and, consequently, is highly sensitive to system noise, as illustrated by subcases a and b (Fig. 1 and Table 1). Qualitatively, however, all perturbations (*Q*, *T_s_*, and σ*_y_*) result in similar behaviors, over similar time frames. These results illustrate that regime changes operate over, and require, billion-year timescales.

### Linking tectonic evolution and oscillations to mantle outgassing and the atmosphere

**Fig. 2.**
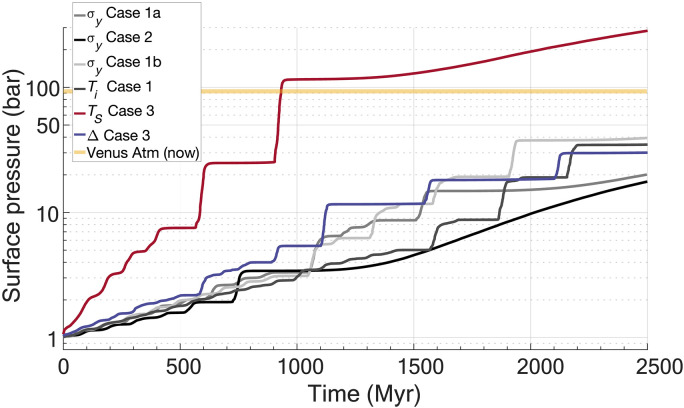
Surface pressure evolution as a function of regime transition for selected results. The current Venusian atmospheric pressure is indicated by the orange line. Cases follow from Table 1.

A planet’s atmosphere is established through the cumulative volcanic outgassing of the interior. In our experiments, we link the evolution of Venus’ atmosphere to its interior through melt production and pressure-dependent volcanic degassing chemistry models [see Methods and (*12*) for details] (*40*). Our initial atmosphere (at time 0 in our experiments) is fixed at 1 bar, consistent with the “habitable” scenario in (*14*). All our cases exploring tectonic transitions generate substantial atmospheres for Venus, ranging from 18 to 281 bars (Fig. 2).

Early plate tectonic–like mobile lid stages of planetary evolution show an approximately linear increase in surface pressures from quasi-steady outgassing rates (Fig. 2). Higher initial surface temperatures (*T_s_* Cases) and internal heating rates (*T_i_* Cases) generate melt more readily and are indicated by initially steeper slopes. As the system destabilizes, magmatic events associated with major mantle overturn phases develop and result in punctuated episodes of atmosphere outgassing. For low surface temperature cases such as for an increase of 30 K (T_s_ Case 1a, fig. S1; Table 1) and decreasing internal heating rates (∆ Cases; Table 1), each overturn generates ~3 to 5 bars of surface pressure. For higher *T_s_* Cases, the atmosphere generated from outgassing during an individual overturn can range up to a substantial ~90 bars for *T_s_* Case 3 (∆*T_s_* of 150 K at ~900 Myr in Fig. 2), with lower values of *T_s_* generating 6 to 20 bars (fig. S1). In all cases, multibar pressures can be generated over 60- to 100-Myr timescales. Following these rapid events, melting and outgassing is subdued for ~300 to 1000 Myr before additional lithosphere breaking events occur or the system enters its final quiescent stagnant lid state. After the final yielding event and the “fallow” period of melt generation, the system thermally equilibrates to its new stagnant lid state, and both melting and surface pressures increase again. This fallow period of low melt and outgassing post-overturn may be effectively indistinguishable from a transition to a stagnant lid if the observation window is short enough (e.g., within the post-overturn phase itself).

All cases considered can generate substantial atmospheric pressures of variable compositions (explored in greater detail in the following paragraph) over the experimental timescale of ~2.5 Gyr, ranging from 19% of Venus’ current atmospheric pressure for the highest yield case (σ*_y_* Case 2) to 306% for a 150-K increase in surface temperatures (*T_s_* Case 3) (Table 1). The mean outgassed atmospheric pressure for all numerical experiments is ~58 bars or ~63% of the currently observed Venusian atmospheric pressure. Experiments with the same control parameters (yield strength, surface temperature, and internal temperature) but slightly differing starting conditions show stochastic variations in overturns and, consequently, atmospheric generation, with a variance of ~20% of Venus’ current atmospheric pressure or ~18 bars. This indicates that the stochastic nature of lithosphere breaking events is at least as important to the generation of an outgassed atmosphere as the weakest atmosphere generating case examined here, which is coincident with the highest standard yield case and, consequently, shortest transition time (Figs. 1 and 2).

**Fig. 3.**
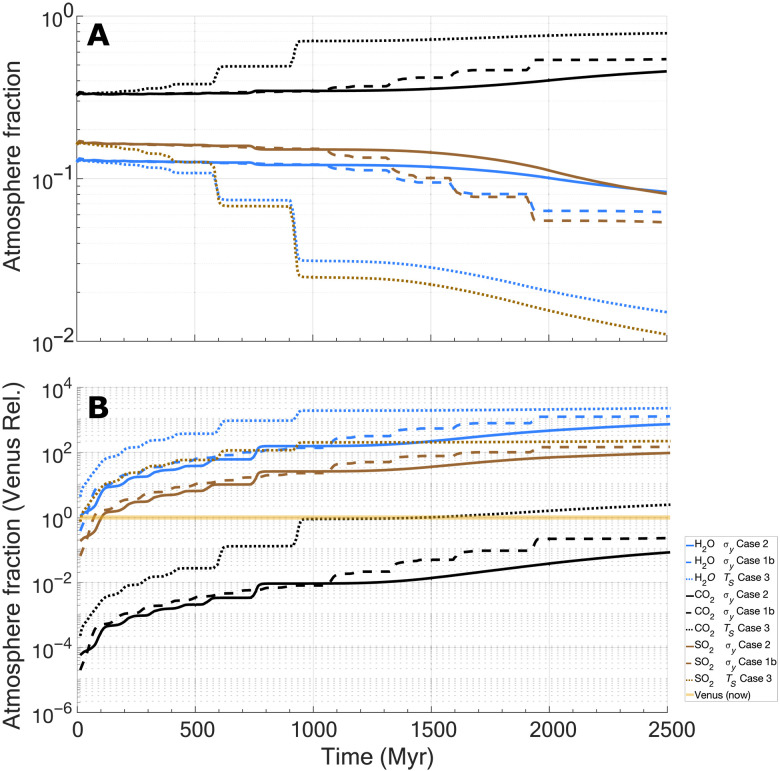
Evolution of chemical species in the atmosphere from outgassing as a function of a regime transition for selected results. Outgassing of H_2_O (blue lines), CO_2_ (black lines), SO_2_ (brown lines) species for (**A**) relative atmospheric fractions and (**B**) abundancies normalized to current Venusian levels (1 indicates observed Venus concentrations; orange bar). For simplicity, current H_2_O in the atmosphere is taken as 20 ppm for this analysis, but abundancies may range from ~0 (no detection) to 100 ppm, with some values approaching ~1350 ppm (these high values may reflect data skewed by trapped sulfuric acid droplets) (*49*). CO_2_ is taken as 96.5% of the bulk Venusian atmosphere but may range by on average by ±0.8% (*49*). SO_2_ is taken as 150 ppm in the bulk Venusian atmosphere but may generally vary between ~38 and <300 ppm (*49*). In general, results above the Venus concentration line indicate potential overproduction in the atmosphere (based on bulk value outlined above), whereas cases below the line indicate potential underproduction. Three representative cases are shown. Cases follow from Table 1.

As surface pressure changes, the outgassing speciation would also be expected to change due to the solubilities of key gases in melt having differing dependences on atmospheric pressure (*40*). Figure 3A illustrates changing atmospheric compositions of select species (CO_2_, H_2_O, and SO_2_) for a representative selection of numerical experiments. Initially, CO_2_ makes up ~34% of the outgassed atmosphere, whereas H_2_O and SO_2_ together make up ~22% (the remainder is made up of trace gases and the starting atmosphere). As the system begins its overturn phase, pronounced shifts in relative fractions of gas species can be seen, with CO_2_ becoming significantly more dominant in the atmosphere and both H_2_O and SO_2_ contributions becoming small but nonzero. The atmospheric outgassing composition can be considered relatively dynamic, with rapid changes up to ~50 bars, after which the speciation curves flatten, and outgassing from additional overturns would compositionally reflect the atmosphere. Although we can generate a considerable portion of Venusian atmospheric CO_2_ in our results, both H_2_O and SO_2_ are seemingly substantially overestimated from bulk atmospheric measurements (see Fig. 3 caption) (*49*) by up to several orders of magnitude (Fig. 3B). For example, with sulfur, an individual overturn can range from ~5 millibars to a substantial 320 millibars of SO_2_ generated per overturn event of ~39 to 60 Myr, which follows from differing melt production rates and changing atmospheric conditions. This is often far in excess of ~13.9 millibars of SO_2_ in the Venusian atmosphere currently [assuming 150–parts per million (ppm) concentrations]. However, isotopic evidence [e.g., (*41*)] shows that Venus has lost substantial amounts of water from its atmosphere, which could reduce or even eliminate the apparent discrepancy between modeled water outgassing and the observed abundance. Furthermore, reactions between the atmosphere and surface can remove SO_2_ from the atmosphere on timescales as short as 10 Myr [e.g., (*50*)], again reducing this potential mismatch.

**Fig. 4.**
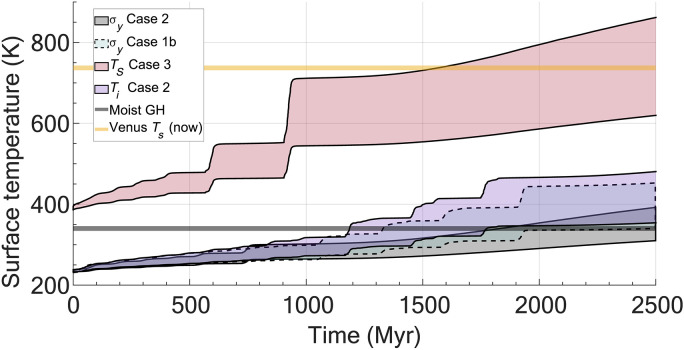
Outgassing surface temperature evolution for select cases as a function of CO_2_, H_2_O, and stellar evolution for Venus. The Venusian albedo is set to 0.7 at a distance of 0.72 au. Surface temperature is calculated from (*51*). The lower bound of the shaded region indicated a water poor atmosphere, whereas the higher bound indicates a water-saturated atmosphere. The shaded regions indicate intermediate atmospheric water concentrations. The moist greenhouse limit (moist GH) is indicated with the gray bar, and the current Venusian surface temperature is indicated by the orange bar. Cases follow from Table 1.

An important effect of punctuated atmospheric generation events is the outgassing of potent greenhouse gases, here for simplicity assumed to be limited to CO_2_ and H_2_O. Surface temperatures are calculated following (*51*), which is a good first-order approximation [based on first-order physics and the Arrhenius relationship between partial pressure of CO_2_ (*P*co_2_), H_2_O, and *T_s_*; see Methods] of the effects of greenhouse forcing. Temperatures may follow two parameterizations: one that is a function of *P*co_2_ alone, giving a lower bound on temperatures (bottom of shaded regions of Fig. 4), and one as a function of both *P*co_2_ and H_2_O (which assumes water saturation of the atmosphere), giving an upper bound on the temperatures (top of shaded regions of Fig. 4). Consequently, the shaded regions themselves reflect varying levels of atmospheric H_2_O concentrations. Starting temperatures are calculated at 0.72 au (astronomical units), with temperature offsets appropriate for each numerical experiment conditions (*T_s_* variations in the starting condition). The evolution of albedo is unconstrained for Venus; we therefore use the current Venusian albedo that is fixed at 0.7 for simplicity. Surface temperature changes are due to increasing solar luminosity and outgassing of greenhouse gases, the former of which accounts for ~10 K over 2.5 Gyr [calculated using the Stefan-Boltzmann relationships and standard stellar luminosity curves (*52*)]. The increase in surface temperature due to stellar luminosity evolution over 2.5 Gyr is less than the smallest surface temperature perturbation found to destabilize the convective system from its precursor state. This indicates that changes in surface temperature over this timescale by luminosity evolution may be insufficient alone to drive tectonic regime shifts. Instead, the contribution and evolution of greenhouse gases may be required to generate sufficient perturbations, as argued in (*46*).

Initially, for most experiments, despite outgassing in a plate tectonic–like mobile lid, greenhouse forcing of surface temperature is of nearly equal importance to increasing luminosity (e.g., approximately a few kelvin). As the system destabilizes, however, each overturn generates punctuated increases of O (10) K in surface temperatures from outgassing for most cases. A notable exception to this is the moderate to high surface temperature conditions for *T_s_* Case 3, which indicates considerable surface temperature increases of O (50 to 100) K. After an overturn, a period of steady surface temperatures develops until either the next overturn or thermal equilibrium in a stagnant lid. Subsequent overturn events, as few as two to three, are sufficient to reach the qualitative runaway greenhouse limit in which liquid water would no longer be stable at the surface of the planet. It is important to note here that the runaway greenhouse limit may not be tied to a specific surface temperature but instead to an insolation rate [e.g., (*53*)]. Other factors such as ocean/landmass distributions and rotation rates may be of equal or, in some cases, greater importance in influencing climate and surface temperature evolution [e.g., (*14*, *31*)]. For this work, we consider the surface temperature range of ~340 to 350 K to be the moist greenhouse limit [e.g., (*54*)]. This approach is not a true runaway state as it is not strictly tied to levels of solar insolation [e.g., (*53*)], but instead, it is due to high atmospheric optical thickness related to increasing greenhouse gas concentrations. In the moist greenhouse state, rapid water loss is thought to occur due to water vapor mixing in the stratosphere and subsequent hydrogen escape to space (a processes we do not consider here in our outgassing experiments) [e.g., (*54*, *55*)]. In these scenarios, water should be considered an upper bound on what could be provided to the atmosphere and, consequently, can offer a prediction of the amount of water lost to space. All cases that start below the moist greenhouse limit/water loss temperature do reach this limit within 1.9 Gyr for water-saturated conditions. However, some dry cases do not reach the moist greenhouse limit by the end of the model at ~2.5 Gyr. Qualitatively, holding all else equal, these results suggest that, for appreciable water contents in the atmosphere, as would be expected from these numerical outgassing experiments, a transition in tectonic regimes can plausibly cause Venus to enter an extreme moist greenhouse state where enhanced water loss would be expected.

## DISCUSSION

### Implications for Venus

Observations of Venus, with a thick atmosphere, interpretations of currently low (relative to Earth), but nonzero, rates of geologically recent volcanism (*32*–*34*), and prodigious volcanism in the past billion years, are consistent with models of planet that is undergoing, or has undergone, a transition in tectonic regimes. Consequently, exploring the linked evolution of the atmosphere, surface, tectonics, and interior may be critical to understanding observations of Venus today. Although several prior studies have explored volcanic outgassing on Venus driven by mantle convection (*12*, *15*, *56*–*58*), these past studies were either limited to a single convective regime or considered the Venusian atmosphere largely to be emplaced before the beginning of the experiment. This study couples and explores outgassing to the dynamically driven, spatially, and temporally complex transition between 3D spherical plate tectonic–like mobile and single-plate stagnant lid convection on Venus. There are, of course, other potential tectonic regimes that may exist [e.g., (*59*)] that are worth exploring, but the focus of this work is on the transition path between end-member early mobile lid and later-stage stagnant lid regimes.

Expanding on the work in (*11*), a small perturbation in surface temperature and global fault strength could serve to destabilize an early epoch of plate tectonic–like mobile lid convection. Once Venus’ tectonic state is destabilized, both Venus’ surface and atmosphere would experience rapid and punctuated changes. Melt production ranges over six orders of magnitude during overturn phases, from a maximum O (10^7^) km^3^/year to a minimum of O (10) km^3^/year. This range encompasses all melt produced in the uppermost mantle of the numerical experiments. The estimated volcanic rates of Venus [e.g., (*32*, *60*)] are generally matched for post-transition periods (Fig. 1) under the constraint of ~10 to 20% melt emplaced volcanically (*56*, *61*–*63*). The characteristics of each individual overturn are somewhat variable, but melt production curves suggest that an individual overturn can emplace a volcanic global equivalent layer of a few percent up to ~10% of the planetary surface, assuming a 1-km-thick uniform distribution, suggesting that ~7 to 8 overturn events (taking the largest producing events) can potentially resurface ~75% of the planet. Assuming thinner layer equivalencies requires fewer hemispheric to subhemispheric-scale overturn events to resurface most of the planetary surface. This indicates that both extensive resurfacing events and current Venusian volcanic rates may be plausibly generated and, as expected, syn- and post-overturn, respectively.

From stochastic large-scale melting events associated with a transition in tectonic regimes, substantial outgassed atmospheres can be generated. During an individual overturn, surface pressure may increase as much as 5 to 10 bars from clement conditions and up to ~90 bars from early elevated surface temperature conditions, over short timescales of ~60 to 100 Myr. With multiple overturn events, outgassed atmospheres of O (10 to 100) bars are plausibly generated in less than several billion years, largely in agreement with observations of Venus today.

Although clearly important, geochemical sinks and atmospheric loss mechanisms [e.g., (*2*, *9*, *12*, *16*)] and their time evolution are largely controlled by their specific geologic, tectonic, atmospheric, and climactic histories, making them largely unconstrained over time for Venus. Given this uncertainty, we consider outgassing and the prior atmosphere (*14*) to be the sole constituents of our modeled atmosphere. For example, we neglect carbon silicate weathering (*51*) and instead assume that outgassed CO_2_ feeds directly into the atmosphere as apparently Venus has followed this path in its recent history. Our results also appear to overpredict both H_2_O and SO_2_ (e.g., Fig. 3B) in the atmosphere. However, both species are reactive in the Venusian atmosphere and would be expected to react with the surface and be sequestered in alteration mineral formation [e.g., (*50*, *64*)]. The results presented here then should be considered an upper end-member of what could be degassed and supplied to the atmosphere, or availability of species, and not necessarily total atmospheric constituents, which will be strongly influenced by the specific history and associated processes of the planet.

**Fig. 5.**
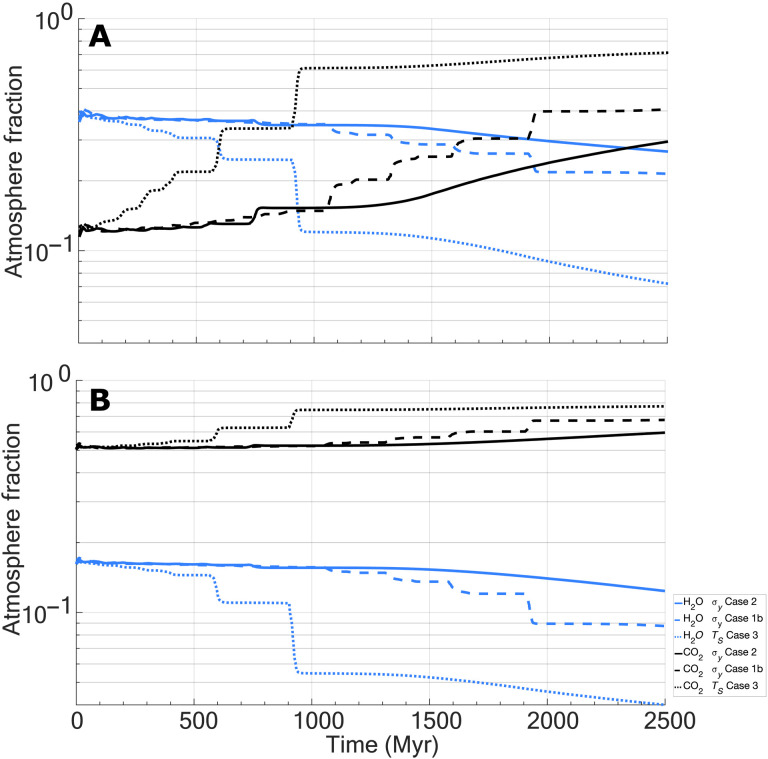
Evolution of chemical species in the atmosphere from outgassing as a function of a regime transition for differing initial atmospheric pressures and H_2_O concentrations. Outgassing and relative atmospheric fractions for H_2_O (blue lines) and CO_2_ (black lines) species following Fig. 3 description for (**A**) 800-ppm H_2_O in the melt and 1-bar initial surface pressure and (**B**) 800-ppm H_2_O in the melt and 50-bar initial surface pressure. Three representative cases follow from Table 1.

An interesting implication of outgassing a substantial atmosphere is that of time variable outgassing speciation and potential atmospheric compositions. For pressures of ~1 bar assuming Earth-like bulk compositions, H_2_O would normally be expected to be the dominant outgassed species [e.g., (*40*)]. However, our models with limited (100 ppm) water in the mantle source region indicate that CO_2_ dominates outgassing. Increasing concentrations of water in the mantle leads to greater outgassing of water; at 1 bar, ~300 ppm of water in the mantle source region is the crossover threshold for water dominance as an outgassed species. Unlike the 100-ppm cases before, assuming that maximal H_2_O concentrations of 800 ppm result in outgassing dominance of H_2_O (Fig. 5A). Despite high H_2_O concentrations, subsequent overturns result in punctuated changes in speciation with CO_2_ becoming dominant by the end of each numerical experiment. Increasing the assumed initial atmosphere pressure to 50 bars with 800 ppm of water broadly resembles the nominal 100-ppm cases (Fig. 3A), with a slightly stronger CO_2_ dominance due to the higher pressure (Fig. 5B). This indicates that increasing water concentrations in the mantle can somewhat offset the reduction in outgassing speciation predicted from the effects of increasing surface pressures. These results suggest that if Venus built its atmosphere from an Earth-like precursor state, then this initial outgassing and atmospheric composition (and surface temperatures) would be highly sensitive to water concentrations in the mantle. However, as the atmosphere builds, the composition would be increasingly insensitive (to perhaps all but the highest water concentration cases). Although outgassed H_2_O concentrations decrease with time (both as an atmospheric and outgassing constituent), they are still appreciable in all the numerical experiments considered, indicating that H_2_O should be still outgassing on Venus today, perhaps at detectable levels. Furthermore, if Venus is outgassing H_2_O currently, then this would be predicted to only be a fraction of the water available in the melt, indicating that solidifying melt would also contain water in hydrous phases (at the time of melt emplacement).

As with all computational models, there are physics that are not explicitly explored in this model space. A more detailed discussion of omitted effects can be found in (*44*). For example, our model considers density changes within the mantle as a function of buoyancy (thermal) terms and does not explicitly consider chemical changes or phase changes of materials. We acknowledge that these effects may shift the absolute quantitative results (e.g., timing of overturn events). They, however, are not considered to be the dominant factors that affect parameters such as surface stress (*65*), which will help serve to control the tectonic regime. It would be beneficial to consider effects such as phase changes in future approaches to understand what effects, if any, they would have on these results.

Our approach in this paper is to outline what a transition in tectonic regimes from a more Earth-like initial state, plate tectonic–like mobile lid and roughly Earth-like and clement surface temperatures to a (eventual) stagnant lid–like Venus would look like and if this would be consistent with the Venus observed at present. We have not considered equilibrium models of volcanic resurfacing that require Venus to be in a single-plate stagnant lid–like state over time [e.g., (*10*, *66*)]. Venus is imperfectly understood, and to date, observations and data cannot rule out either model suite. Of interest, however, is that the stagnant lid results presented here are not necessarily completely inconsistent with equilibrium models. Melt production in stagnant lid simulations sufficiently after a transition in tectonic states is enhanced relative to plate tectonic–like mobile lid baseline rate (e.g., Fig. 1), in agreement with the requirement that equilibrium models have higher than normal (relative to Venus at present) volcanic flux [e.g., (*10*)]. This prediction holds for a stagnant lid state that transitions ~500 Myr to ~1 Gyr ago. However, if “recent” volcanic rates are as low as inferred [e.g., (*32*, *60*)], then our model offers a tantalizing prediction: Venus recently underwent an overturn (within the past few 100 Myr) and that volcanism on Venus is at or near minima and will increase with time into the future.

We have shown that Venus’ atmosphere composition, pressure, and surface temperature can be natural consequences of a transitioning planet, which has interesting implications and caveats for the Venusian record. Although both the generation of a thick outgassed atmosphere and extensive resurfacing events are potentially the result of the same stochastic processes, their evolution and sampling potential may be critically different. For example, surface samples may only record the last stages of a large-scale event (e.g., surface lavas and flows). Surface lavas can then be considered to sample a discrete system state that may be wildly out of sync with the current (and near future) system state (e.g., Fig. 1). In contrast, the bulk atmosphere (predominantly here CO_2_) samples the time-integrated specific evolution of the coupled interior, surface, and exosphere and, consequently, may not reflect the most recent melting events or planetary states. The bulk atmosphere is then generated and modified over billion-year timescales, whereas melting and surface flows reflect, at most, ~60- to 100-Myr intervals. Consequently, direct observations and potential sampling (e.g., lava flows and units) may be a very poor indicator of the generation and evolution of the cumulative atmosphere (let alone mantle state) and may instead only readily indicate the last vestiges of the last large-scale melting event. A caveat to this may lie in water and sulfur cycling, which may be required to maintain a global cloud deck. In this sense, although the bulk atmosphere may be insensitive to small/recent volcanism, it has a sensitivity to much shorter timescales [e.g., (*38*)] and may serve as a strong indicator of active volcanism. Overturn events in our models suggest that if Venus was Earth-like in surface temperature at the start of a transition in tectonics, then it would require ~1 to 2 Gyr to reach the moist greenhouse limit, which would facilitate rapid water loss (assuming a water-saturated atmosphere). This would imply that the current Venusian atmosphere, and consequently its water loss, is quite recent (geologically speaking). Results both in Fig. 1 and in (*11*) indicate that the transition from a mobile lid to a fully stagnant lid can take several billion years, so Venus may well still be in the midst of such a transition. If so, our results here indicate that its atmospheric composition and pressure and its surface temperature might well continue to undergo additional punctuated changes in the future.

Forthcoming robotic missions to Venus by both the NASA and European Space Agency may help clarify the tectonic and atmospheric evolution of Venus. Mass spectrometry measurements of the isotopic composition and total atmospheric abundance of noble gases, nitrogen, and hydrogen by the DAVINCI mission are expected to provide important new insights into the history of water in the atmosphere, the geologically recent and long-term volcanic outgassing rates, and loss processes from the atmosphere (*67*), all of which can help test the model predictions made here. High spatial resolution topography and radar imagery by the EnVision and VERITAS missions will help constrain the style and magnitude of surface deformation at various points in Venus history (*68*), thus testing the basic hypothesis of a transition from plate tectonic–like mobile lid mantle convection to single-plate stagnant lid convection.

## METHODS

To model global, thermal-tectonic convection, we use the well-benchmarked open-source software CitcomS [e.g., (*69*–*71*)], which solves the dimensionless governing equations of mass, momentum, and energy conservation, assuming infinite Prandtl number and Boussinesq approximations, in full 3D spherical geometries. This approach neglects the effects of mantle compressibility, phase changes, and depth-dependent thermal expansivity and conductivities. These simplifications are made to focus on the physics of an evolving tectonic state and to avoid an intractably large parameter space in this work. The nondimensional forms of the governing equations for conservation of mass, momentum, and energy are given in the convention of Einstein summation by

$$u_{i,j}=0$$ (1)

$$-P_{,i}+{\left[\eta \left(u_{i,j}+u_{j,i}\right)\right]}_{,j}+\mathrm{RaT}\delta_{\mathrm{ir}}=0$$ (2)

$$T_{,t}+u_{i}T_{,i}=T_{,\mathrm{ii}}+Q$$ (3)

where u is the velocity vector, P is the pressure, η is the reference viscosity, *Ra* is the Rayleigh number, *T* is the temperature, $\delta_{\mathrm{ir}}$ is the Kronecker delta (subscript *r* denotes radial direction), *Q* is the internal heat production, and *t* is the time. The subscripts *i* and *j* represent the spatial indices, and $X_{,y}$ represents the derivative of *X* with respect to *y*.

The vigor of the convective system is described by a thermal Rayleigh number (*Ra*)

$$\mathrm{Ra}=g\rho \alpha \Delta Td^{3}/\left(\kappa \eta \right)$$ (4)

which is defined for pure basal convection, where *g* is gravity, α is thermal expansivity, ρ is density, κ is the thermal diffusivity, ∆*T* is the reference temperature drop given as the temperature contrast from the convecting layer depth (*d*), or base, to the surface (*T_b_ − T_s_*). The internal heat generation rate is given through the nondimensional quantity *Q*

$$Q=Hd^{2}/\left(\kappa \Delta T\right)$$ (5)

where *H* is the radioactive heating rate. The internal heating rate *Q* typically is shown to be linked to the basal *Ra* through the input internal heating *Ra* (*Ra_Q_*) as: *Ra_Q_* = Q*Ra.

Simulations used here include a temperature-dependent viscosity formulation

$$\eta \left(T\right)=\eta_{l}\text{exp}\left(A\left[\frac{1}{T+1}-\frac{1}{2}\right]\right)$$ (6)

where η*_l_* is a preexponential factor and *A* is the nondimensional activation energy, which controls the temperature-dependent viscosity contrast. Besides viscous flow, rock near the surface is expected to plastically yield, which can be described by a Byerlee (*72*)–type yield stress-strain relationship parameterized as a Drucker-Prager or Mohr-Coulomb type of criterion within a viscous formulation [e.g., (*73*, *74*)].

CitcomS uses temperature- and depth-dependent viscosity and plastic yielding formulations to simulate planetary-scale tectonics (*75*, *76*). At stresses below the failure criteria, the ductile flow law of the mantle follows a temperature-dependent branch (Eq. 6), at the yield criterion the flow law operates within the plastic branch with an effective yield viscosity given as

$$\eta_{\text{yield}}=\frac{\sigma_{y}}{I}$$ (7)

where *I* is the second strain-rate invariant and σ*_y_* is the nondimensional yield strength. We consider yielding at the cohesive limit ($\tau_{0}$) of the lithosphere (i.e., strength of the lithosphere at zero hydrostatic pressure), which nondimensionally is related to σ*_y_* as

$$\sigma_{y}=\left(\frac{d^{2}}{\kappa \eta_{0}}\right)\tau_{0}$$ (8)

where $\eta_{0}$ is the viscosity of the lithosphere. Plastic yielding formulations allow for localization of failure zone within the lithosphere, which are analogous to weak plate boundaries on the Earth.

For our experiments, we use a mantle *Ra* of 6 × 10^5^ with a temperature-dependent viscosity contrast of 6 × 10^4^ (*A* set to 22.0042 in Eq. 6). The viscosity of the mantle is reduced by a factor of 30 in the upper 20% of the domain and is enhanced by a factor of 10 in the upper 10% of the mantle [see (*11*) for more details], simulating a low-viscosity upper mantle and high-viscosity lithosphere. The exact viscosity profile of Venus is uncertain at present; however, if Venus once operated in a plate tectonic–like mobile lid state, it might be expected to exhibit a reduced low-viscosity upper mantle [e.g., (*11*)]. The inclusion of both a high-viscosity and intrinsically strong lithosphere and a low-viscosity asthenosphere-like zone allows for a total possible viscosity contrast between the upper mantle and surface of 1.8 × 10^7^. Boundary conditions are free slip, and the basal temperature is fixed at a nondimensional value of unity. The core to planetary radius ratio is fixed at 0.55, and each of the 12 spherical caps contains 33 by 33 by 33 nodes (393,216 elements), which has been shown to be sufficient to model these types of problems (*11*, *45*). The initial thermal state is taken from a robust mobile lid state with high levels of internal heating to emulate a planetary system early in its evolution, such that *Ra_Q_* ~ 10^7^ for most cases, which are thought to be roughly equivalent to the *Ra* of planetary mantles [e.g., (*10*) and references therein]. Each experiment is thermally dependent on precedent system states with key planetary parameters (*Q*, *T_s_*, and σ*_y_*) varied, independently or in tandem, to destabilize the system and usher in a change in tectonic regimes [see (*44*, *77*) for more details]. Each experiment is run two to three times longer than required to reach a quasi-statistically steady state to ensure system stability. Additional parameters follow (*11*). Melt production is calculated in a postprocessing step using well-established solidus and liquidus curves for peridotite (*78*) [see (*11*) for additional details]. This approach allows us to calculate a globally averaged melt production rate over time.

We link the evolution of Venus’ atmosphere to its interior through melt production and pressure-dependent volcanic degassing chemistry models (*40*) fixed to a bulk silicate Earth (BSE) compositional constraint (*79*). The outgassed species released from volcanism are dependent on atmospheric surface pressure evolution and oxygen fugacity (*f*O_2_) of the mantle. The redox conditions of the mantle are fixed at FMQ-1.5 (the fayalite-magnetite-quartz buffer) (*40*), which is consistent with both the average *f*O_2_ of basalts (*80*) measured on differentiated bodies in our Solar System and the experiments that suggest that the *f*O_2_ of the Earth’s mantle to be largely fixed after late-stage core formation [e.g., (*81*); see (*12*) for additional references]. We therefore assume surface pressures to dominate gas speciation in these results. The model in (*40*) assumes basaltic volcanism to be the primary volcanism on the planetary body and that volatile species are outgassed under the constraint of experimentally constrained equilibrium between the melt and gas phases. This work uses the well-established equilibrium constants and mass balance method (*82*), which determines C-O-H-N-S equilibria [see (*40*) and references therein for more details].

The concentration of water in the Earth’s mantle, let alone the Venusian mantle, is unknown, ranging from a nominally dry ~5 to 50 ppm to a relatively water-rich ~800 ppm (*41*, *83*–*85*). We choose an initially partially dry condition with 100 ppm of water and appropriate bulk partition coefficients (*86*). Consistent with BSE, we assume that the bulk solid state Venusian mantle contains 600 ppm C (*87*, *88*) and 800 ppm S [Earth’s upper mantle averages ~400 ppm, whereas basaltic melts may be enriched up to 1000 ppm (*89*); 800 ppm is taken here as our nominal value]. For our experiments, melt available to interact with the atmosphere is restricted to 10% of the total melt formed following commonly used intrusive/extrusive ratios (*56*, *61*–*63*). The atmosphere here is a function of the initial atmosphere taken from (*14*) as a starting condition, melt production over time as a function of tectonic state, and volcanic speciation of melt as a function of evolving surface pressures over time and tectonic states. For simplicity, regassing, the release of volatile species from metamorphic reactions, and surficial-atmospheric chemical reactions are not considered.

To explore surface temperatures, we use the approach in (*51*) to link CO_2_ levels with *T_s_*, with and without H_2_O saturation. A simple approximation for the combined effects of CO_2_ and H_2_O on temperature follows

$$T_{s}=T_{s}^{*}+2\left(T_{e}-T_{e}^{*}\right)+4.6{\left(\frac{{P\text{CO}}_{2}}{{P\text{CO}}_{2}^{*}}\right)}^{0.346}-4.6$$ (9)

with forcing by CO_2_ alone

$$T_{s}=T_{e}+2.3{\left(\frac{{P\text{CO}}_{2}}{{P\text{CO}}_{2}^{*}}\right)}^{0.346}$$ (10)

where $T_{s}^{*}$ is the temperature of the surface, $T_{e}$ is the effective temperature, $T_{e}^{*}$ is the effective temperature at the start of the simulation, and the ratio $\frac{{P\text{CO}}_{2}}{{P\text{CO}}_{2}^{*}}$ is the change in CO_2_ pressures overtime. The effective temperature is calculated following

$$T_{e}={\left[\frac{S\left(1-A\right)}{4\sigma }\right]}^{0.25}$$ (11)

where *S* is the solar flux at the top of the atmosphere at 0.72 au and increasing stellar luminosity with time, consistent with the evolution of a star along the Hertzsprung-Russell main sequence (which can be found in any standard astronomy textbook), A is the albedo (assumed to be 0.7), and σ is the Stefan-Boltzmann constant.
