# Supplementary material for: The punctuated evolution of the Venusian atmosphere from a transition in mantle convective style and volcanic outgassing
Source: Sci Adv. 2025 Jan 10;11(2):eadn9861. doi: 10.1126/sciadv.adn9861 (PMC11721565; doi:10.1126/sciadv.adn9861)
Supplement: Supplementary file 1 — Fig. S1 [file sciadv.adn9861_sm.pdf]

Science Advances
AAAS

Supplementary Materials for

The punctuated evolution of the Venusian atmosphere from a transition in mantle convective style and volcanic outgassing

Matthew B. Weller and Walter S. Kiefer

Corresponding author: Matthew B. Weller, wellem@rpi.edu

Sci. Adv. **11**, eadn9861 (2025)
DOI: 10.1126/sciadv.adn9861

This PDF file includes:

Fig. S1

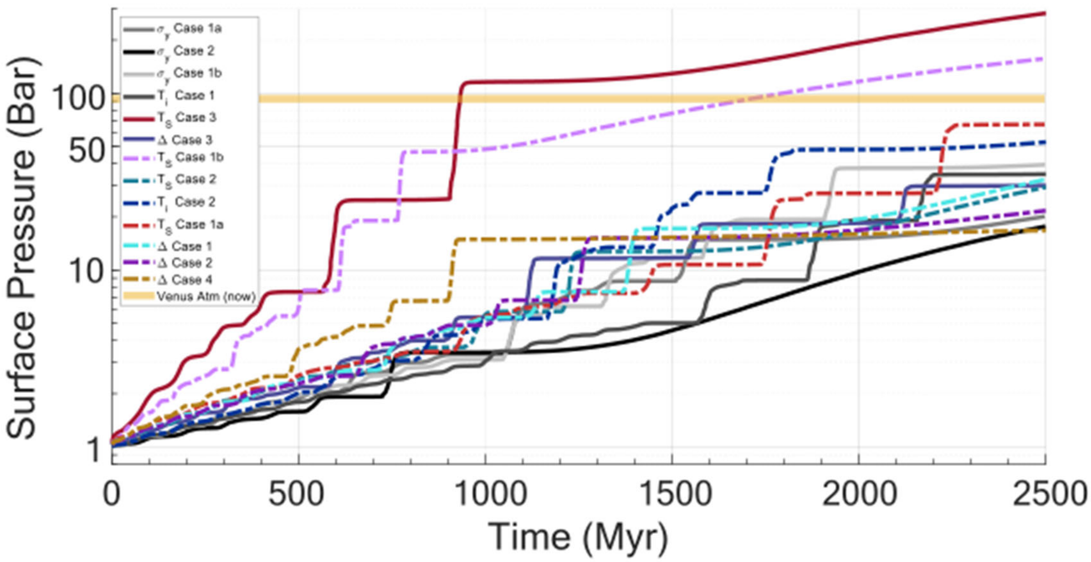

Fig. S1. Surface pressure evolution as a function of regime transition for all model results. The current Venusian atmospheric pressure is indicated by the orange line. All cases follow from Table 1.
